# Recent advances in large-scale protein interactome mapping

**DOI:** 10.12688/f1000research.7629.1

**Published:** 2016-04-29

**Authors:** Virja Mehta, Laura Trinkle-Mulcahy

**Affiliations:** 1 Department of Cellular and Molecular Medicine, Ottawa Institute of Systems Biology, University of Ottawa, Ottawa, ON, Canada

**Keywords:** Interactome, Proteomics, AP-MS, affinity purification-mass spectrometry, XL-MS, cross-linking MS analysis, PCP, protein correlation profiling, BioID, APEX

## Abstract

Protein-protein interactions (PPIs) underlie most, if not all, cellular functions. The comprehensive mapping of these complex networks of stable and transient associations thus remains a key goal, both for systems biology-based initiatives (where it can be combined with other ‘omics’ data to gain a better understanding of functional pathways and networks) and for focused biological studies. Despite the significant challenges of such an undertaking, major strides have been made over the past few years. They include improvements in the computation prediction of PPIs and the literature curation of low-throughput studies of specific protein complexes, but also an increase in the deposition of high-quality data from non-biased high-throughput experimental PPI mapping strategies into publicly available databases.

A range of complementary approaches are currently being used to identify protein-protein interactions (PPIs) in a large-scale, high-throughput manner ( Figure 1). These include affinity purification-mass spectrometry (AP-MS), cross-linking MS (XL-MS) analysis, MS-based protein correlation profiling (PCP), and yeast two-hybrid (Y2H) screens. Proximity labeling techniques, based on the identification (by AP-MS) of near neighbor proteins by spatially restricted enzymes, can also be used to map protein networks and probe complex structures, although they have yet to be applied at a whole proteome level. In this review, we discuss recent applications of these diverse methods to large-scale protein interactome mapping and the public availability of the resulting datasets for both high-throughput bioinformatic analysis of protein interaction networks and single-protein information for more focused studies.

**Figure 1.**
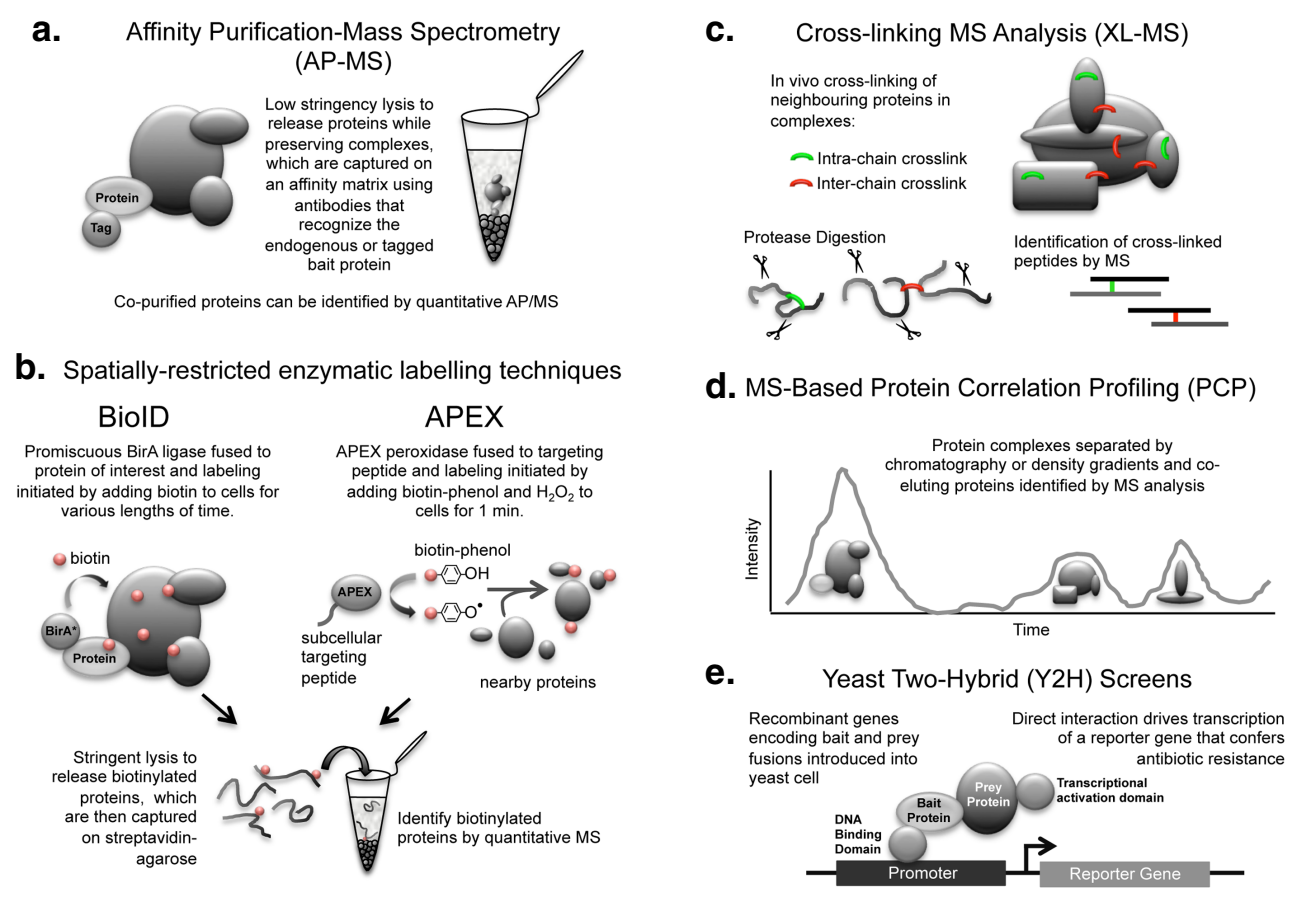
Examples of experimental approaches used to map protein-protein interactions. **a**. Affinity purification-mass spectrometry approach for identifying proteins that associate with a particular bait protein. **b**. Two spatially-restricted “near neighbor labeling” approaches that utilize enzymatic reactions to tag proteins (for capture and identification) that associate with a bait protein. **c**. Strategy behind cross-linking mass spectrometry analysis of multiprotein complexes. **d**. Protein correlation profiling approach for identifying multiprotein complex members that co-elute following various separation techniques. **e**. Strategy behind the classic yeast two-hybrid method used to screen for direct protein-protein interactions.

## Affinity purification-mass spectrometry-based large-scale protein-protein interaction mapping initiatives

Currently, the most popular strategy for both high- and low-throughput interactome mapping is AP-MS, in which an endogenous or tagged bait protein is depleted from cell lysates by using an affinity resin and associated proteins identified by liquid chromatography-tandem mass spectrometry (LC-MS/MS) ( Figure 1a). Two recent large-scale studies of human PPIs used AP-MS approaches to identify more than 20,000 interactions, respectively ( Table 1). To assemble what they call the BioPlex (biophysical interactions of ORFeome-derived complexes), Huttlin and colleagues C-terminally FLAG-HA tagged about 600 human open reading frames (ORFs) and transiently overexpressed them in HEK293T cells, identifying co-precipitating proteins by AP-MS ^1^. Clone validation, quality control, inclusion of positive and negative controls, and development of a quantitation algorithm (CompPASS-Plus) based on abundance, detection frequency, and reproducibility were employed to increase confidence in the resulting dataset, which was deposited into the BioGRID PPI database last year. The authors consider this to be phase 1 of their long-term effort to map interactomes for the entire human ORFeome collection and are continuing to post updates that can be freely browsed or downloaded from their website.

**Table 1.** Recent large-scale interactome screens using a variety of protein-protein interaction mapping techniques. AP-MS, affinity purification-mass spectrometry; BAC, bacterial artificial chromosome; CORUM, Comprehensive Resource of Mammalian protein complexes; GFP, green fluorescent protein; LC-MS/MS, liquid chromatography-tandem mass spectrometry; MS, mass spectrometry; ORF, open reading frame; XL-MS; cross-linking mass spectrometry; Y2H, yeast two-hybrid.

Approach	System	Coverage	Dataset Availability	Reference
AP-MS experiments identifying proteins that co-precipitate with GFP-tagged bait proteins	N- and C-terminally tagged mouse and human BAC transgenes stably integrated in HeLa cells	28,500 interactions involving 5,400 proteins	Deposited into IntAct: http://www.ebi.ac.uk/intact and the IMEx consortium: http://www.imexconsortium.org	2
AP-MS experiments identifying proteins that co-precipitate with FLAG- HA-tagged bait proteins	C-terminally FLAG- HA-tagged ORFs in ORFEOME collection v8.1 transiently overexpressed in HEK293T cells	23,744 interactions involving 7,668 proteins	Deposited into BioGRID: http://thebiogrid.org Updates can be browsed or downloaded at: http://gygi.med.harvard.edu/projects/bioplex	1
XL-MS study utilizing MS-cleavable cross-linkers combined with sequential CID-ETD-MS/MS acquisition and XlinkX search engine	HeLa cell lysates	2,179 unique cross-links detected (1,665 intraprotein and 514 intraprotein)	Reported in Supplementary Data and raw files available as project #890 here: https://chorusproject.org XlinkX publically available: http://sourceforgenet/project/xlinkx/	38
Yeast 2-hybrid screens	>15,000 human ORFs from hORFeome v5.1	~14,000 high-quality human binary protein-protein interactions	Data (published and updated) can be browsed at: http://interactome.dfci.harvard.edu	45
Native size-exclusion chromatography combined with LC-MS/MS	U2OS cell lysates	>8,000 proteins identified and 1,061 of 1,970 CORUM complexes mapped	Data available at: www.peptracker.com/encyclopediaInformation/	41
Biochemical fractionation combined with quantitative MS profiling	HeLa S3 and HEK293 cell lysates	5,584 proteins identified and 622 putative protein complexes described	Data deposited into BioGRID: http://thebiogrid.org and publicly accessible here: http://human.med.utoronto.ca	42
Size-exclusion chromatography and MS-based protein correlation profiling	HeLa cell lysates	7,209 binary interactions clustered into 291 protein complexes	All IDs reported in Supplementary Data and scripts used for analysis available here: http://www.chibi.ubc.ca/faculty/foster/software/	43

The approach used by Hein and colleagues ^2^ involved screening a library of 1,125 HeLa cell lines with stably incorporated N- and C-terminally tagged mouse and human bacterial artificial chromosome (BAC) transgenes under near endogenous control ^3^ by AP-MS, as demonstrated previously in focused studies analyzing chromosome segregation ^4^ and the function of motor proteins ^5^. In addition to identifying more than 28,000 interactions in their large-scale screen, the authors estimated interaction stoichiometries (based on absolute quantitation of protein abundances in complexes and compared for both N- and C-terminally tagged and mouse and human bait proteins) and measured the relative cellular abundances of interaction partners. An interesting finding was the predominance of weak (i.e., sub-stoichiometric) interactions in the global interactome, which may suggest that stable complexes rely on weak links to connect to each other and to transient/dynamic regulators. The interaction datasets were submitted to both the IntAct database and the IMEx consortium.

Importantly, both studies demonstrated significant overlap with the CORUM (Comprehensive Resource of Mammalian protein complexes) database, a manually curated repository of more than 2,800 mammalian protein complexes ^6^. CORUM is currently considered the “gold standard” PPI database because it is based solely on high-confidence, experimentally verified interactions and does not accept deposition of large-scale datasets ( Table 2). Proteome coverage was also high for both studies, as assessed by comparison with datasets generated and shared in recent large-scale whole proteome mapping initiatives ( Table 3) such as the MaxQuant Database ^7– 9^ (MaxQB), the Human Proteome Map ^10^, and ProteomicsDB ^11^.

**Table 2.** Examples of online protein-protein interaction databases.

Database	Description	Link
CORUM	Manually curated repository of experimentally characterized protein complexes high-throughput experiments excluded)	http://mips.helmholtz-muenchen.de/genre/ proj/corum/
MIntAct	Open-source, open data molecular interaction database (merger of IntAct and MINT databases) curated from literature and from direct date depositions	http://www.ebi.ac.uk/intact
The BioGRID Interaction Database	~750,000 non-redundant interactions drawn from >55,000 publications for 30 model organisms	http://thebiogrid.org
IMEx Consortium	Common curation platform for 11 molecular interaction databases	http://www.imexconsortium.org/
Complex Portal	Open-source, manually curated resource to collate protein complexes from >10 major model organisms	http://www.ebi.ac.uk/intact/complex

**Table 3.** Recent large-scale whole proteome mapping initiatives.

Database	Description	Link
Human Proteome Map	Proteome data from 30 human tissue samples (17 adult and 7 fetal); 6 purified haematopoietic cells); Proteins encoded by 17,294 genes identified (~84% of total annotated)	http://www.humanproteomemap.org
ProteomicsDB	Combined data available from repositories and contributed by colleagues, representing 60 human tissues, 147 cell lines, 13 body fluids; Coverage for 18,097 of 19,629 human genes	https://www.proteomicsdb.org
MaxQB	Proteome data from 11 different human cell lines (19,865 total proteins; average 10,361 ± 120 proteins per cell line and other model organisms)	http://maxqb.biochem.mpg.de/mxdb/

Although the standard caveats of AP-MS strategies still apply, namely the potential for overexpression or tag-induced artefacts and the predominance of false positives such as non-specific background proteins ^12– 14^ and the recently described cryptic protein binding to cloning regions or “scars” where affinity tags are linked to the gene of interest ^15^, these large-scale studies benefit tremendously from the comparison of multiple experiments. Negative controls are largely bait-independent, and thus common contaminants are highlighted by their appearance in numerous unrelated datasets. Moving forward, the limitations of AP-MS can be further minimized by a variety of strategies, including direct affinity tagging of endogenous proteins using the powerful CRISPR/Cas9 (clustered regularly interspaced short palindromic repeats/Cas9) gene editing tool ^16, 17^, more rigorous assessment of the quality and specificity of antibodies used to capture endogenous proteins for AP-MS ^18^, and improvements in significance analysis software ^19, 20^.

## Proximity-based labeling strategies

Although AP-MS remains the most commonly used technique for mapping PPIs, its Achilles heel has always been the necessity to break cells open to extract complexes for analysis, which can be disruptive to the underlying PPIs and hinder identification of weak or transient associations or both. The development of complementary proximity labeling approaches that use spatially restricted enzymes to biotinylate neighboring proteins has helped to address this key issue. Complex members are labeled covalently *in vivo*, thus eliminating the need for low-stringency purification strategies to preserve their integrity. Furthermore, the high affinity of streptavidin for biotin facilitates efficient recovery of biotinylated proteins from lysates for MS analysis.

Two particular proximity labeling techniques, BioID and APEX, have been employed recently for the analysis of multiprotein complexes and for identification of the protein components of specific cellular compartments ( Figure 1b). BioID involves expression of a protein of interest fused to a prokaryotic biotin ligase and the subsequent biotinylation of amine groups on neighboring proteins when excess biotin is added to the cells. Whereas the wild-type BirA biotin ligase from *Escherichia coli* is capable of transferring biotin only to a substrate bearing a specific recognition sequence, the generation of a promiscuous BirA (Arg118Gly mutant) permits the biotinylation of any protein found within a 10-nm labeling radius ^21, 22^. As with AP-MS, identification of a protein-protein association using BioID does not imply a direct physical interaction.

BioID has enabled the identification of proteins involved in important functional complexes that were previously difficult to characterize because of the limitations of AP-MS. For example, the identification of ubiquitin ligase substrates by AP-MS is challenging and this is due in part to the weak and transient interactions observed between the ligase and its substrates. A BioID approach, however, facilitated identification of novel substrates ^23^. This type of approach has also been used to identify novel c-MYC ^24^ and HIV-1 Gag ^25^ interacting partners, highlight force-dependent molecular interactions at cell-cell adhesions ^26^, identify proteins localized to cell junction complexes ^27, 28^ and the centrosome-cilium interface ^29^, and probe the structure of the centrosome ^30, 31^ and the nuclear pore complex ^22^.

APEX is a monomeric peroxidase reporter derived from pea ^32^ or soybean ^33^ ascorbate peroxidase that catalyzes the oxidation of biotin-phenol to biotin-phenoxyl in the presence of H _2_O _2_, resulting in the biotinylation of proteins in the neighboring region. Whereas BirA-catalyzed biotinylation is limited to Lys residues, biotin-phenoxyl radicals can covalently react with electron-rich amino acids such as Tyr, Trp, His, and Cys. They are also short-lived (<5 ms) and membrane-impermeable and have a small labeling radius (<20 nm). APEX can also catalyze diaminobenzidine precipitation to generate contrast after OsO _4_ fixation, which allows confirmation of localization at nanometer resolution by electron microscopy ^32^. A second-generation APEX2 (Ala134Pro mutant) with improved efficiency was shown to function even better as both a promiscuous labeling enzyme and an EM tag ^34^. Similar to BioID, once proximity labeling has been achieved, biotinylated proteins can be identified via stringent streptavidin purification and MS analysis. An advantage of APEX over BioID is higher temporal resolution, as labeling is achieved on a minute rather than an hour scale.

The APEX reporter has been used to map the proteome of the human mitochondrial intermembrane space and membrane-enclosed mitochondrial matrix ^33, 35^, the *Drosophila* muscle mitochondrial matrix proteome ^36^, and the proteome of the cilium ^37^. Although the applicability of APEX to interactome mapping out with membrane-bound organelles has not yet been demonstrated, further optimization of the enzyme and substrate could extend its utility.

## Large-scale protein-protein interaction mapping initiatives based on alternative approaches

High-quality large-scale interactome datasets have also been assembled using strategies such as XL-MS, which provides additional information about the topographical structure of protein complexes ( Figure 1c and Table 1). In the case of XL-MS, progress was initially slowed by the complexity of data acquisition and analysis, in particular the two overlapping series of fragment ions from each peptide that appear in the MS/MS spectrum. Although major advances have been made ^38, 39^, including the development of MS-cleavable cross-linkers that fragment efficiently in the MS/MS mode to yield two major fragment ions corresponding to the component peptides (which can be subsequently identified by MS ^3^), sensitivity can be further improved in the future by the addition of pre-fractionation steps, the use of affinity-tagged cross-linking agents or complementary chemistry (i.e., agents that cross-link amino acids other than lysine ^40^), digestion with complementary proteases, and the development of dedicated software for the analysis of complex XL-MS datasets.

Similarly, PCP-MS studies ( Figure 1d) also continue to increase in coverage and specificity, comparing favorably to reference interactome datasets ^41– 43^. This approach avoids affinity purification steps and instead separates and maps protein complexes using a variety of approaches that include density gradients and size-exclusion, ion-exclusion, and hydrophobicity interaction chromatography. Given the range of separation options available, PCP-MS also offers significant scope for advancement in the future.

## Large-scale binary protein-protein interaction mapping

Although XL-MS does identify direct protein interactions, the other approaches discussed above (AP-MS, proximity labeling, and PCP-MS) can confirm only that proteins exist in the same multiprotein complex. A complementary technique that has been used for more than 20 years to detect direct PPI is the Y2H assay. In this approach, the bait and prey proteins are tagged to the DNA binding and transcriptional activation domains of a split transcription factor, and direct binding drives its reconstitution and subsequent activation of a reporter gene ( Figure 1e). Although limited by technical and biological challenges that include the need to construct large libraries and the high false-negative and -positive rates that arise from the absence of certain post-translational modifications in yeast that govern protein-protein associations in mammalian cells and forced interactions that do not occur in mammalian cells under physiological conditions, the Y2H screen remains a powerful approach for detecting or confirming (or both) binary interactions.

Using the extensive human ORF collection as bait/prey in an ongoing series of large-scale Y2H screens, researchers at the Dana-Farber Cancer Institute in Boston are addressing the question of which PPIs in the human interactome are direct ^44, 45^. With the long-term goal of mapping the full range of human binary PPIs, their most recent update added about 14,000 new binary interactions, bringing the current total to about 17,000. The full dataset, and future updates, can be browsed using their dedicated web interface ( Table 1).

## Conclusions

With a daunting grand plan for these PPI network maps to comprehensively characterize individual protein functions and global proteome organization, it is not surprising that significant challenges remain. As noted above, the stringency and efficiency of protein extraction and depletion remain an issue with AP-MS studies, and traditional mapping strategies still favor the most abundant/robust interactors. It is hoped that, as complementary approaches such as proximity labeling, XL-MS, and PCP-MS increase in sensitivity and specificity, they will provide extended coverage of the interactome. Importantly, as more high-quality large-scale datasets are collected and shared via online interaction databases like MIntAct ^46^ and BioGRID ^47^ ( Table 2), consistencies and patterns will emerge.

Additional technical challenges, posed by their hydrophobic nature, have particularly hampered the identification of PPIs among membrane proteins (and between membrane proteins and soluble proteins such as cytosolic signaling factors). However, the success of recent large-scale initiatives such as the mapping of more than 12,000 binary interactions between *Arabidopsis* membrane/signaling proteins using the mating-based split ubiquitin system (mbSUS) in yeast ^48^ and the TAP (tandem affinity purification)-MS based screening of 1,590 putative budding yeast membrane proteins using three different mild, non-denaturing detergent purification strategies in parallel ^49^ (1,726 PPIs and 501 putative heteromeric complexes identified) demonstrates that these challenges are also surmountable.

Other challenges include the necessity to define PPIs over a wider range of biological contexts, given that some are cell cycle- or developmental stage-specific, for example, or occur only under particular physiological conditions or in response to specific post-translational modifications. An ambitious future goal is a comprehensive and quantitative high-throughput approach that combines gene-editing with live super-resolution imaging and interactome mapping to define the dynamic localization, composition, and topography of functional multiprotein complexes.

## Abbreviations

AP-MS, affinity purification-mass spectrometry; CORUM, Comprehensive Resource of Mammalian protein complexes; ORF, open reading frame; PCP, protein correlation profiling; PPI, protein-protein interaction; XL-MS; cross-linking mass spectrometry; Y2H, yeast two-hybrid.
